# Long-range correlation-guided dual-encoder fusion network for medical images

**DOI:** 10.1038/s41598-025-22834-1

**Published:** 2025-11-06

**Authors:** Tao Zhou, Zhe Zhang, Huiling Lu, Mingzhe Zhang, Jiaqi Wang, Qitao Liu

**Affiliations:** 1 School of Computer Science and Engineering, North Minzu University, Yinchuan, 750021 China; 2 Key Laboratory of Image and Graphics Intelligent Processing of State Ethnic Affairs Commission, North Minzu University, Yinchuan, 750021 China; 3 School of Medical Information and Engineering, Ningxia Medical University, Yinchuan, 750004 China

**Keywords:** Deep learning, Dense network, Long-range correlation coefficient, Medical image fusion, Multi-scale features, Biological techniques, Computational biology and bioinformatics

## Abstract

Multimodal medical image fusion plays an important role in clinical applications. However, multimodal medical image fusion methods ignore the feature dependence among modals, and the feature fusion ability with different granularity is not strong. A Long-Range Correlation-Guided Dual-Encoder Fusion Network for Medical Images is proposed in this paper. The main innovations of this paper are as follows: Firstly, A Cross-dimension Multi-scale Feature Extraction Module (CMFEM) is designed in the encoder, by extracting multi-scale features and aggregating coarse-to-fine features, the model realizes fine-grained feature enhancement in different modalities. Secondly, a Long-range Correlation Fusion Module (LCFM) is designed, by calculating the long-range correlation coefficient between local features and global features, the same granularity features are fused by the long-range correlation fusion module. long-range dependencies between modalities are captured by the model, and different granularity features are aggregated. Finally, this paper is validated on clinical multimodal lung medical image dataset and brain medical data dataset. On the lung medical image dataset, IE, AG, ${\textbf {Q}}^{{\textbf {AB/F}}}$, and EI metrics are improved by 4.53%, 4.10%, 6.19%, and 6.62% respectively. On the brain medical image dataset, SF, VIF, and ${\textbf {Q}}^{{\textbf {AB/F}}}$ metrics are improved by 3.88%, 15.71%, and 7.99% respectively. This model realizes better fusion performance, which plays an important role in the fusion of multimodal medical images.

## Introduction

Multimodal medical image fusion is to fuse medical images of different modals into one image, which provides a more comprehensive technical support for disease diagnosis and treatment. Medical images play an important role in computer-aided detection and diagnosis of malignant tumors. However, due to the difference of medical imaging equipment, different modals medical images examine different characteristics of the human body, and a single modality of medical images does not provide sufficient information. For example, Computed Tomography (CT) clearly displays bones and high-density structures information, CT images provide limited information on organ metabolism. Positron emission tomography (PET) reflects biological metabolic processes and neurotransmitter activity, but its spatial resolution is low. Magnetic resonance imaging (MRI) has advantages in imaging human soft tissue, but it does not reflect metabolic activity. Multi-modal medical image fusion aims to provide reliable references for clinical diagnosis and scientific research by integrating complementary and redundant information from images of different modalities^1^, which assists doctors in accurately diagnosing lesions^2^.

In recent years, deep learning is a key technology in multimodal medical image fusion^3^. The fusion methods are generally classified into 3 categories: Convolutional Neural Network (CNN)-based fusion methods, Autoencoder (AE)-based fusion methods, and Generative Adversarial Network (GAN)-based fusion methods. CNN-based fusion methods are a technology that uses convolutional neural network to extract and fuse image features. It learns local features through the convolutional layer and reduces the feature dimension through the pooling operation, and finally realizes the fusion with different modal image. Tang^4^ et al. proposes the Residual Decoder-Encoder Detail-Preserving Cross Network (DPCN), which employs a dual-branch framework to extract structural details from the source image. However, because the model only uses the last layer results, it is easy to lose the information of the middle layer. Umirzakova^5^ et al. propose a spatial/channel dual attention CNN combined with deep learning reconstruction (DLR), which improves the feature extraction. VIF-Net^6^ adopts a hybrid loss function that combines a modified structural similarity metric and total variation, it adaptively fuses thermal radiation and texture details through unsupervised learning. Image fusion methods based on encoder–decoder networks obtain fused images by designing and training encoders and decoders. The encoder extracts features, and the decoder reconstructs them, effectively mitigating the network depth impact on performance. DenseFuse^7^ introduces a dense connection mechanism in the encoder, effectively resolving the intermediate layer information loss issue and achieving better fusion results. Res2Net^8^ integrates ResNet into the encoder, enhancing the network’s multi-scale feature extraction capacity. GAN-based fusion methods use adversarial learning between the generator and discriminator to estimate the target probability distribution, thereby implicitly performing feature extraction, feature fusion, and image reconstruction. DSAGAN^9^ uses a dual-stream structure and multi-scale convolutions to extract deep features, thus enhancing the fused features with an attention mechanism to generate the final fused image. UCP^2^-ACGAN^10^ presents an adaptive conditional GAN model that uses a context perceptual processor to obtain context perceptual feature maps, which better highlight the lesion regions in the fused image. Zhou et al.^11^ propose a GAN model with dual discriminators, which uses the source image’s semantic information as constraints to generate semantically consistent images. However, multimodal medical image fusion still faces several challenges: In the encoding phase, existing methods don’t achieve effective interaction among different modalities and different granular features. In the feature fusion, the internal dependencies between modality are ignored in some degree, and it is difficult to capture the long-range dependencies between local and global features effectively. To solve this problem, this paper proposes a Long-Range Correlation-Guided Dual-Encoder Fusion Network for Medical Images. The main contributions of this paper are as follows:

- A Long-Range Correlation-Guided Dual-Encoder Fusion Network for Medical Images is proposed. In the encoder, a Cross-dimension Multi-scale Feature Extraction Module and a dense network architecture are used to strengthen the feature Transmitting-Reuse ability among different layers. In the fusion module, it uses correlation calculation and layer-by-layer aggregate strategy to capture the long-range dependencies between different modal images.
- Aiming at the effective feature extraction problem at different dimension features. This paper designs Cross-dimension Multi-scale Feature Extraction Module (CMFEM). In the feature extraction stage, multi-scale features are extracted along the height and width dimensions, enhancing the network’s sensitivity for lesion size.
- Aiming at the problem of feature dependence between modalities. In the fusion stage, this paper designs a Long-range Correlation Fusion Module (LCFM). which calculates the long-range correlation coefficient between local features and global features, the features of the same granularity are fused by the LCFM. Long-range dependencies between modalities are captured, and features of different granularity are aggregated, avoiding detail information being neglected.

## Methodology

**Figure 1:**
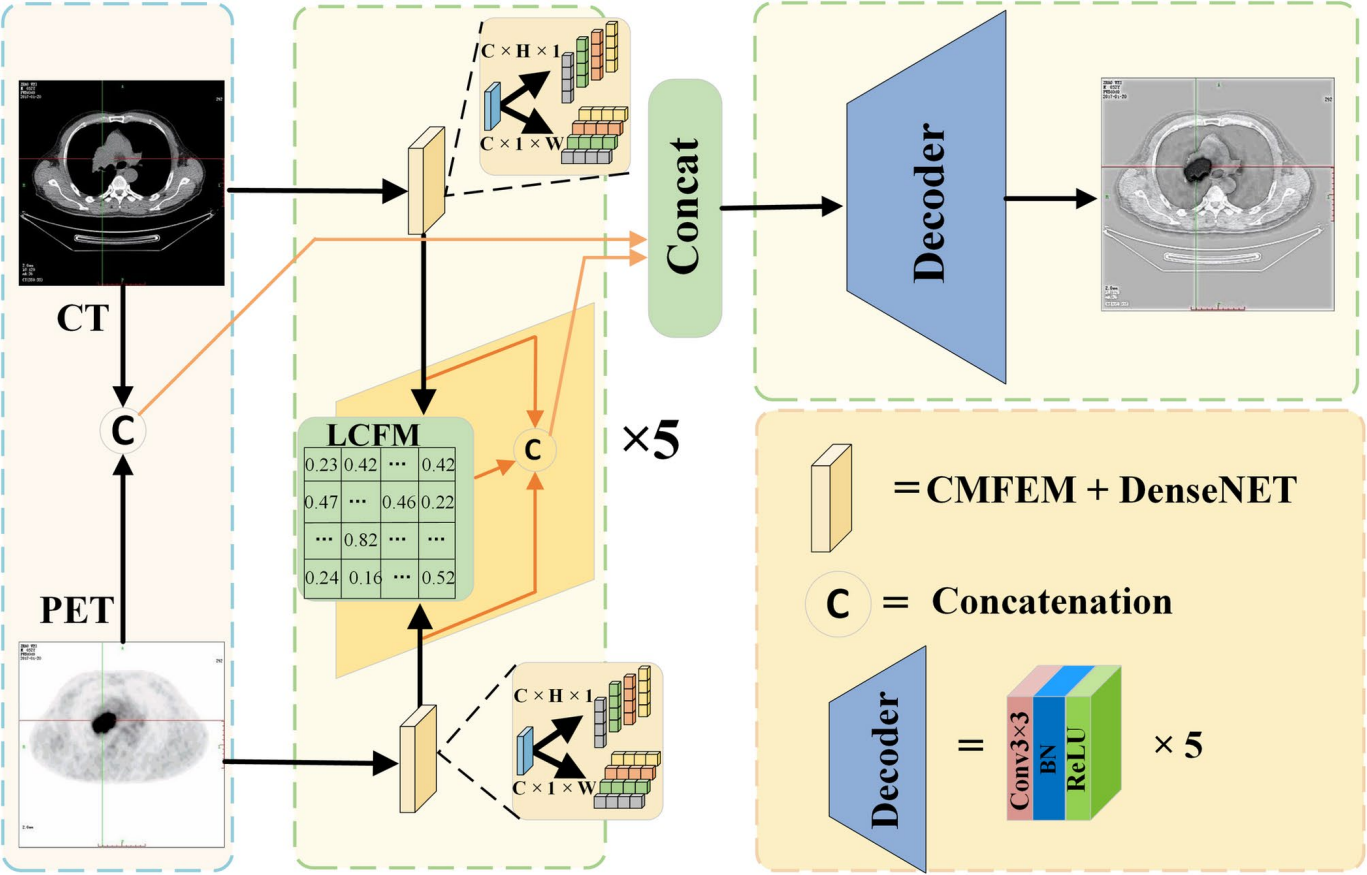
Long-range correlation-guided dual-encoder fusion network.

Existing multimodal medical image fusion methods generally focus on improving the individual modalities’ fine-grained feature extraction ability, but it neglects the inter-modal feature dependencies and the effective fusion about different granularity features. This paper designs a Long-Range Correlation-Guided Dual-Encoder Fusion Network for Medical Images, including the Cross-dimension Multi-scale Feature Extraction Module (CMFEM), Long-range Correlation Fusion Module (LCFM), and the loss function construction. The Long-Range Correlation-Guided Dual-Encoder Fusion Network for Medical Images adopts a dual-branch network structure, and it extracts coarse-to-fine grained features from the two modes through the dense connection structure, which enables efficient feature extraction and fusion. Each branch includes 5 feature extraction layers, where the 1 to 4 layers consist of 4 CMFEM blocks, and the last layer uses a 1$\times$1 convolution followed by Tanh as the nonlinear activation function. To reduce information loss, inspirated by DenseNet, this paper uses dense connections on each branch, which strengthen the feature transmitting-reuse ability among different layers. In order to improve the interaction ability of multi-scale features, the extracted image features of each layer are fused by LCFM module, and the fused features are concatenated and aggregated to generate global fused images. Finally, the model reconstructs the image using five 3$\times$3 convolution layers, generating a fusion image with sharp edges and clear lesion regions. The network structure of the Long-Range Correlation-Guided Dual-Encoder Fusion Network for Medical Images is shown in Fig. 1.

### Cross-dimension multi-scale feature extraction module

**Figure 2:**
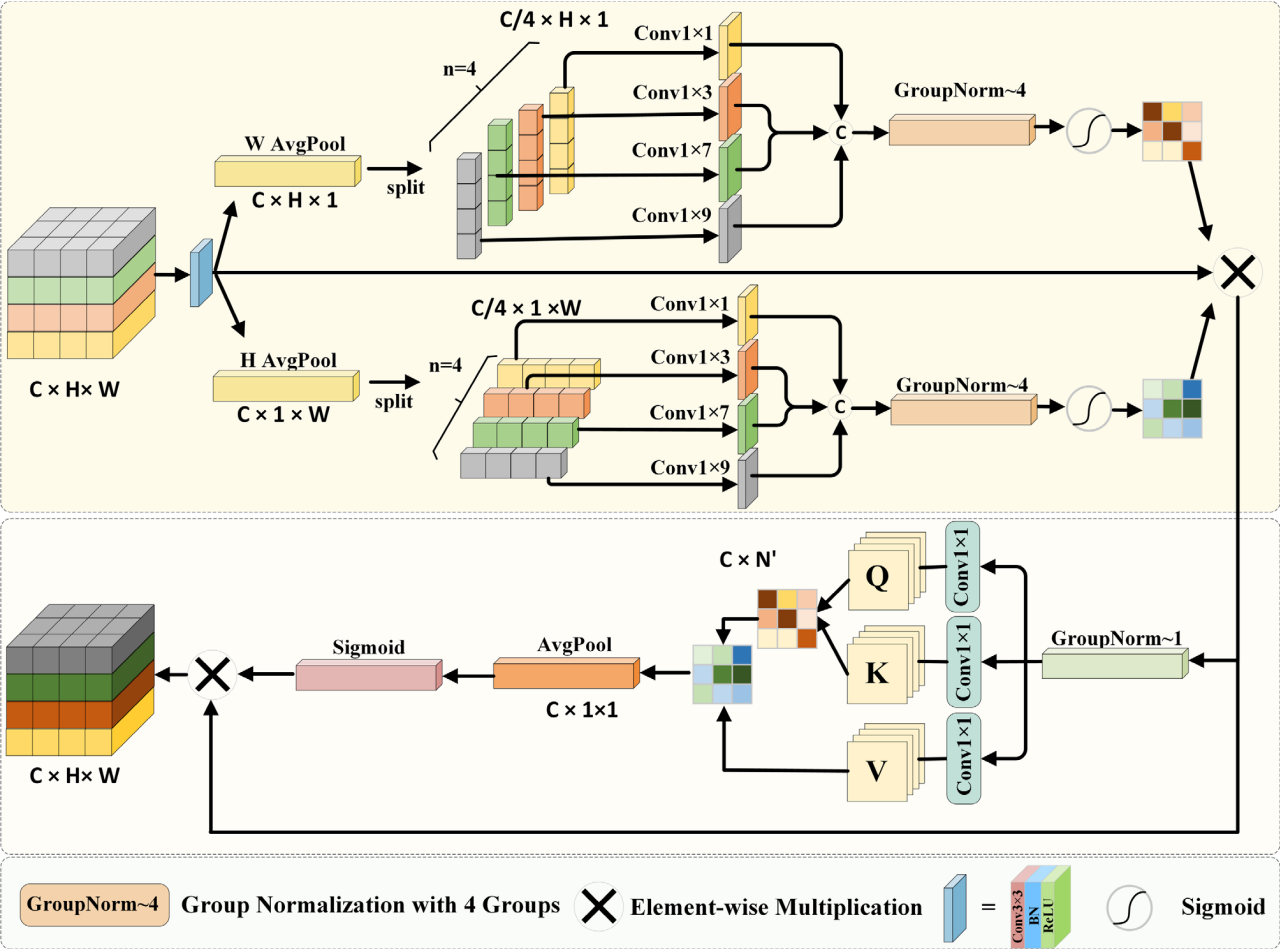
Cross-dimension multi-scale feature extraction module.

Attention mechanism is a technique that simulates the ability of human visual attention and is used in deep learning to help models focus on important parts of input images. models are able to be more efficient and precise in handling complex tasks. A model weight is a parameter used to adjust the importance of input features. the weights determine how much each feature influences the final output. Channel attention deals with the relationship between channels in the image. Its core idea is to evaluate which channels are more important for the current task and dynamically adjust the activation intensity of each channel accordingly. Spatial attention mechanism focuses on the spatial dimension of the image, which enhances the model’s ability to focus on specific areas by applying weights to different positions of the input images. For multimodal medical images, spatial information is reflected as semantic features at the pixel level, where local spatial information is helpful to capture fine-grained low-level semantic features, and global spatial information supports the recognition and understanding of high-level semantic features. Due to the complexity of multimodal medical images, a single attention mechanism struggles to achieve extract critical features. Therefore, this paper designs a Cross-dimension Multi-scale Feature Extraction Module (CMFEM), as shown in Fig. 2, which extracts features of different scales through multi-scale convolution in width and height dimensions to obtain multi-scale feature Xs. Then, it computes the self-attention in space, enhances the features of the spatial information, and obtains the channel attention. This approach not only helps to reduce information redundancy and data complexity, but also improves model performance, making feature extraction of PET, CT and MRI images more accurate and efficient.

As shown in Fig. 2, the internal structure of Cross-dimension Multi-scale Feature Extraction Module (CMFEM), Firstly, the input $X\in {\mathbb {R}}^{B\times C\times H\times W}$ is convolved and decomposed along the height and width dimensions. Two one-dimensional sequences are created by using global average pooling: $X_H\in {\mathbb {R}}^{B\times C\times W}$ and $X_W\in {\mathbb {R}}^{B\times C\times H}$. Secondly, in order to capture spatial information at different scales, the features are split into 4 sub-feature maps, $X_H^i$ and $X_w^i$, where i$\in$ $\{1,2,3,4\}$, each sub-feature map has C/4 channels, it efficiently capture the diverse spatial information within sub-feature maps. The module utilizes one-dimension convolutions with kernel sizes of 3,5,7,and 9 for the 4 sub-feature maps. In addition, in order to solve the issue of limiting receptive fields caused by using one-dimension convolutions, this paper utilizes lightweight sharing convolution. This method captures consistent features between the two dimensions indirectly modeling their dependencies, which expands the perceptive field and improves feature representation ability. Then, 4 groups of Group Normalization (GN) are applied for normalization, followed by a Sigmoid activation function to generate spatial attention, which activates specific spatial regions. Finally, the feature maps $F_H$ and $F_W$ from the H and W dimensions are multiplied with the input feature map *X* to obtain $X_\textrm{s}.$ This process is represented by the following formula (1)–(5):

1 $$\begin{aligned} & X_H^i = \operatorname {Conv1d}_i\Bigl (\operatorname {Pool}_H^{C\rightarrow \frac{C}{4}}\bigl (\operatorname {Conv2d}(X)\bigr )\Bigr ). \end{aligned}$$

2 $$\begin{aligned} & X_W^i = \operatorname {Conv1d}_i\Bigl (\operatorname {Pool}_W^{C\rightarrow \frac{C}{4}}\bigl (\operatorname {Conv2d}(X)\bigr )\Bigr ). \end{aligned}$$

Where *X* represents the input feature map, and $X_H^i$ and $X_W^i$ represent the spatial structural information of the *i*th sub-feature along the H and W directions. ${\hat{i}}$ represents the *i*th sub-feature, ${\hat{i}} \in \{1, 2, 3, 4\}$.

3 $$\begin{aligned} & F_H\quad =\sigma \left( GN_H^4\left( Concat(X_H^1,X_H^2,X_H^3,X_H^4)\right) \right) . \end{aligned}$$

4 $$\begin{aligned} & F_W\quad =\sigma \left( GN_W^4\left( Concat(X_W^1,X_W^2,X_W^3,X_W^4)\right) \right) . \end{aligned}$$

5 $$\begin{aligned} & X_s=F_H\times F_W\times X. \end{aligned}$$

Where $\sigma (\cdot )$ represents the Sigmoid activation function, and $GN_H^4(\cdot )$ and $GN_W^4(\cdot )$ represent the 4 group normalization along the H and W directions, $X_s$ represents the spatial information of X.

In order to retain and utilize the multi-scale spatial information extracted by multi-scale convolution, this paper uses a self-attention module to enhance the spatial prior information, which improves the performance of the model. Firstly, 3 different mapping functions $F_j^Q,F_j^K$, $F_j^V$are used to project $X_s$ into the query, key and value respectively, and Q, K, V are obtained. These features are used in subsequent attention calculations to obtain $X_F$, then, $X_F$ is compressed into one-dimension vector and activated by the Sigmoid function. Finally, the enhanced feature map *F* is obtained by multiplying *X*s with the feature map that is calculated by Sigmoid and average pooling operation. The process is represented by the following formula (6)–(8):

6 $$\begin{aligned} & Q=F_j^Q(X_p),K=F_j^K(X_p),V=F_j^V(X_p). \end{aligned}$$

7 $$\begin{aligned} & X_F=F(Q,K,V)=Softmax\left( \frac{QK^T}{\sqrt{C}}\right) V. \end{aligned}$$

8 $$\begin{aligned} & F=X_s\times \sigma \left( Pool_{(H^{\prime },W^{\prime })}^{(H^{\prime },W^{\prime })\rightarrow (1,1)}(X_F)\right) . \end{aligned}$$

Where $F_{\text {proj}}(\cdot )$ represents the mapping functions for generating the query, key, and value. $\sigma (\cdot )$ represents the Sigmoid activation function, and *F* represents the final output feature map.

### Long-range correlation fusion module

To address the problem of feature dependence between modalities, this paper designs a Long-range Correlation Fusion Model (LCFM). This module captures the long-range dependencies between local and global features by calculating the correlation, and these dependencies are encoded into a correlation matrix. Then, 1$\times$1 convolution layer is used to reduce the dimension of the correlation matrix. After that, the two correlation feature maps are added, their size is compressed to 1$\times$1 by adaptive pooling, and then multiply with the input feature map to enhance the feature representation. In the last layer of the LCFM module, the two enhanced feature maps are concatenated with the input feature map along the channel dimension. The features of the same granularity are fused through the LCFM, which captures the long-range dependencies between the different modalities, and then the features of different granularities are aggregated. This paper presents the forward flow of LCFM in Algorithm 1.

**Figure 3:**
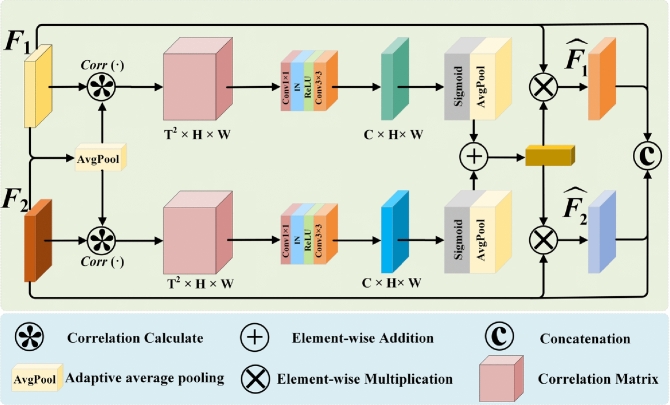
Long-range correlation fusion module.

The structure of the LCFM module is shown in Fig. 3, The LCFM fuses the feature maps extracted by CMFEM. In image fusion tasks, capturing the long-range dependencies of different modalities is the key to image fusion. However, when capturing long-range dependencies, overly relying on global information leads to the loss of fine details, and overly relying on local information fails to capture global semantic relationships. Therefore, balancing the relationship between the two in network design and ensuring that they can work together is a challenge. To solve this issue, in this paper, the long-range dependencies of local and global features are captured by calculating the correlation of different modalities. For example, the correlation between the features $F_{i}$ and $F_{j}$ is calculated by the following formula (9):

9 $$\begin{aligned} Corr(F_i,F_j)=\frac{F_i\cdot F_j}{\parallel F_i\parallel _2^2\parallel F_j\parallel _2^2}. \end{aligned}$$

Where $F_{i,j} \in {\mathbb {R}}^{C \times H \times W}$ ($i, j \in \{1,\ldots ,N\}$), and $\Vert \cdot \Vert _2^2$ represents the L2 norm. Since the two modalities from the same scene are registered, the correlation distribution range remains consistent. The correlation $Corr(F_i, F_j)$ between two different modality images ranges from [-1, 1]. long-range dependencies are captured by correlation calculation, but correlation calculation is complex and time-consuming. For example, when the feature map size is N=H$\times$W, the computational cost is $N^2$. As the feature map size increases, the computational cost becomes extremely large. To overcome this problem, this paper introduces a pooling operation to simplify the calculation by the following formula (10):

10 $$\begin{aligned} {\widehat{F}}_{temp}=AdapAvgPool_{(16,16)}(F). \end{aligned}$$

Where $AdapAvgPool(\cdot )$ is adaptive average pooling, which is used to compress the feature map and generate the feature map ${\hat{F}}_{temp} \in {\mathbb {R}}^{C \times T \times T}$. Then, the feature map ${\hat{F}}_{temp}$ is used to compute the correlation with the original image by the following formula (11):

11 $$\begin{aligned} Corr\left( F^i,{\widehat{F}}_{temp}^k\right) =\frac{F^i\cdot {\widehat{F}}_{temp}^k}{\left\| F^i\right\| _2^2\left\| {\widehat{F}}_{temp}^k\right\| _2^2}. \end{aligned}$$

Where ${\widehat{F}}_{temp}^k\in {\widehat{F}}_{temp},(k\in \{1,\ldots ,{\mathcal {T}}^2\}).$ In the fusion process, $1\times 1$ convolution is used to reduce the dimension and linear transform input features, which enables reduce the redundancy between channels and highlights important features. 3$\times$3 convolution is used to improve the ability of capturing local features. The Sigmoid activation function is used to introduce nonlinearity and constrain the output values to the range of [0,1]. Finally, $Corr_{F_1}$ and $Corr_{F_2}$ are obtained by adaptive average pooling of the feature maps using the following formula (12) and (13).

12 $$\begin{aligned} & \text {Corr}_{F_1} = \text {AdapAvgPool}\Bigl (\sigma \Bigl (\text {Conv}_{3 \times 3}\Bigl (\text {Conv}_{1 \times 1}\Bigl (\text {Corr}_{F_1}^{\text {temp}}\Bigr )\Bigr )\Bigr )\Bigr ). \end{aligned}$$

13 $$\begin{aligned} & \text {Corr}_{F_2} = \text {AdapAvgPool}\Bigl (\sigma \Bigl (\text {Conv}_{3 \times 3}\Bigl (\text {Conv}_{1 \times 1}\Bigl (\text {Corr}_{F_2}^{\text {temp}}\Bigr )\Bigr )\Bigr )\Bigr ). \end{aligned}$$

Where $Corr^{temp}_{F_1}$ and $Corr^{temp}_{F_2}$ represent the long-range correlation matrices of features $F_1$ and $F_2$, respectively. $Conv_{1\times 1}(\cdot )$ represents the 1$\times$1 convolution layer, $Conv_{3\times 3}(\cdot )$ represents the 3$\times$3 convolution layer, $\sigma (\cdot )$ represents the Sigmoid activation function, and $AdapAvgPool(\cdot )$ represents the adaptive average pooling operation. Then, the obtained feature maps are multiplied with the original feature maps by the following formula (14,15) and ():

14 $$\begin{aligned} {\widehat{F}}_1=F_1\otimes Corr_{F_1}.\end{aligned}$$

15 $$\begin{aligned} {\widehat{F}}_2=F_2\otimes Corr_{F_2}. \end{aligned}$$

Finally, the feature maps are concatenated using a concatenation strategy:

16 $$\begin{aligned} {\widehat{F}}_f=concat({F}_1,{F}_2,{\widehat{F}}_1,{\widehat{F}}_2). \end{aligned}$$

where ${\hat{F}}_f$ represents the fused feature, and $concat(\cdot )$ represents the concatenation operation along the channel dimension.

### Loss function

For the medical image fusion task, in this paper, the fusion network is trained in an unsupervised manner. The loss function of the Long-Range Correlation-Guided Dual-Encoder Fusion Network for Medical Images is designed, it is the combination of intensity loss and gradient loss by the following formula (17):

17 $$\begin{aligned} L_{f}=L_{int}+\alpha L_{grad}. \end{aligned}$$

Where $L_f$ represents the total loss, $L_{int}$ represents the intensity loss, $L_{grad}$ represents the gradient loss, $\alpha$ is a hyperparameter.

Intensity Loss: The intensity loss ensures the global brightness consistency of the fused image by constraining the low-frequency components of the image. Therefore, the intensity loss is defined as formula (18):

18 $$\begin{aligned} L_{int}=L_{int}^{F1}+L_{int}^{F2}. \end{aligned}$$

Where $L_{int}^{F1}$ and $L_{int}^{F2}$ represent the intensity loss for the images, which are defined as formulas (19) and (20):

19 $$\begin{aligned} & L_{int}^{F1}=\frac{1}{HW}\parallel I_f-I_{CT}\parallel _1. \end{aligned}$$

20 $$\begin{aligned} & L_{int}^{F2}=\frac{1}{HW}\left\| I_f-I_{PET}\right\| _1. \end{aligned}$$

Where *H* and *W* represent the height and width of the image, and $\Vert \cdot \Vert _1$ represents the $L_I$-norm.

Gradient Loss: The gradient loss is used to capture the high-frequency components of the image to ensure the accurate localization about the lesion and the clarity of the image texture information. Therefore, the gradient loss is defined as formula (21):

21 $$\begin{aligned} L_{grad}=\frac{1}{HW}\parallel \nabla I_f-\max (\nabla I_{F1},\nabla I_{F2})\parallel _1. \end{aligned}$$

Where $|\cdot |$ represents the absolute operation, $\nabla$ represents the image gradient is computed using the Sobel operator, and max($\cdot$) is the operation to obtain the maximum value.

**Algorithm 1:**
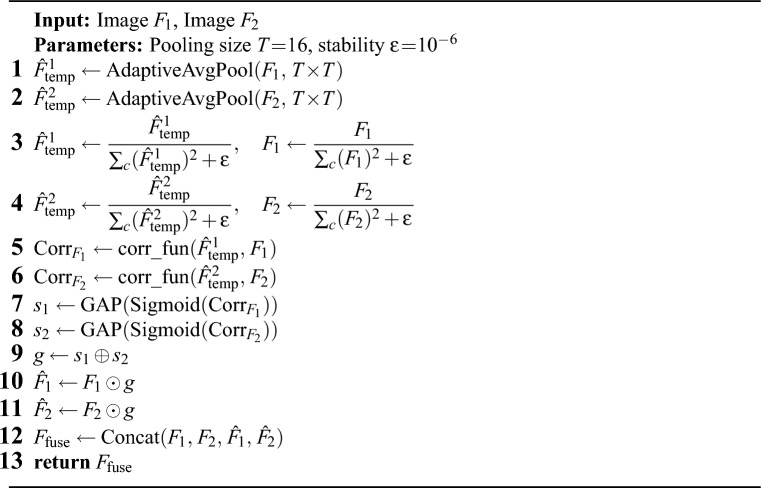
LCFM (forward pass)

## Results

### Dataset

The model is trained on two different datasets:

1. Lung tumor PET/CT images, The dataset uses clinical patients with lung tumors who underwent PET/CT general examination in a top-three hospital in Ningxia from January 2018 to June 2020. These images are high-quality, without artifacts, and clearly show tumor lesions. The patients did not undergo radiofrequency ablation or lung resection, and they have complete and detail pathological reports. The experiment includes 95 patients who met the specified criteria. Among them, There are 46 women (48%) aged between 30 and 80 years, with an average age of 54.32 years. There are 49 male (52%) aged between 27 and 74 years, with an average age of 50 years, and the height of the patients is not restricted. Patients need to do the following preparations before the PET/CT general examination: fast for 6 hours, which ensure that blood sugar is below 10, urinate, and remove metal ornaments. The patient is injected with 3.7mBq/kg deoxyglucose and waited for 1 hour. Subsequently, the patient lies flat in a dark room and waits for 45 to 60 minutes. Then, PET/CT images of the lungs and torso are collected, including cross-sectional, sagittal, and coronal images. To ensure the correct labeling of lesions and ensure the accuracy of the data, the dataset is evaluated and diagnosed by three expert physicians combined with clinical experience. The final result is decided according to the opinion of the majority experts. The three expert doctors include a thoracic surgeon with 8 years of clinical experience, a pulmonologist with 5 years of clinical experience, and a radiologist specializing in radiology. The final number of samples for the two image datasets of different modalities is 2430, respectively. In this paper, 1000 PET images and 1000 CT images are selected as the training set, and 400 are selected as the test set. The labels of the images are manually drawn by two clinicians. The data is transformed into JPG format by algorithms, and the image is adjusted to 356$\times$356 pixels. These pre-processing steps are designed to improve image quality and adapt to the training requirements of neural networks.
2. Brain MRI/PET images. This dataset comes from Harvard Medical dataset. 269 MRI images and 269 PET images are selected from this dataset. In order to expand the training dataset, this paper applies data augmentation to the MR-PET images, which generates 807 MRI images and 807 PET images. In this paper, 600 MRI images and 600 PET images are selected as the training set, and 200 MRI images and 200 PET images are selected as the test set. The size of the training image is 256 $\times$ 256.

### Experimental environment

Random seed: All experiments use a fixed random seed (seed = 42). Data split: Training/validation/test = 74%/10%/16%. Early stopping: Monitored metric = validation QAB/F; patience = 10, min_delta = 1e-4. During training, we use a batch size of 8 and the Adam optimizer. The initial learning rate is 0.01, and it is reduced by 10% every 5 epochs. Training runs for 80 epochs. Hardware Environment: The processor is Inetl(R) Xeon(R) Gold 5218 CPU @ 2.30GHz, Memory: 64GB, GPU: NVIDIA TITAN RTX. Software Environment: Windows Server 2019 Datacenter 64-bit operating system, Pytorch 1.12.1 deep learning framework, Python version 3.7.12, CUDA version 11.3.58.

### Comparison experiment and evaluation metrics

#### Comparison experiment design

In order to verify the effectiveness of the Long-Range Correlation-Guided Dual-Encoder Fusion Network for Medical lmages, two sets of comparative experiments are conducted in the PET/CT image dataset of lung tumors. The first set of experiments is compared with decomposition transformation methods, including method 1: image fusion method based on NSCT^12^. Method 2: LatLRR^13^, the second set of experiments is compared with deep learning methods, including Method 3: Multi-modal image fusion method EMMA^14^. Method 4: Unsupervised DIF-Net^15^ based on encoder–decoder. Method 5: DATFuse^16^. Method 6: Fusion method based on dense Res2net and dual non-local attention model, Res2Fusion^8^. Method 7: U2Fusion^17^. Method 8: GAN-FM^18^. Method 9: CDDFuse^19^. In the brain MRI/PET image dataset, the fusion results of 6 deep learning-based methods are compared. Method 1: CDDFuse^19^; Method 2: DATFuse^16^; Method 3: EMMA^14^; Method 4: MATR^20^; Method 5: U2Fusion^17^; Method 6: PLAFusion^21^; Method 7: DDBFusion^22^; Method 8: MMIF^23^; Method 9: MURF^24^. To ensure the fairness of the comparison, all parameter values of the above methods are set to the default values specified by their authors.

In this paper, 8 evaluation metrics widely used in the field of image fusion are used, including Information Entropy (IE)^25^, Average Gradient (AG)^26^, Standard Deviation (SD)^27^, Spatial Frequency (SF)^28^, Sum of the Correlations of Differences (SCD)^19^, Visual Information Fidelity (VIF)^29^, Edge Preservation Values Q^AB/F^^30^, and Edge Intensity (EI)^31^. Among them, IE is used to measure the randomness or variation of pixel values in an image. AG is used to represent image sharpness, reflecting the richness of texture details in the image. SD is used to measure the degree of variation in pixel values and the difference in brightness, reflecting the image’s contrast and details. SF describes the frequency and periodicity of brightness or color changes at different locations in the image, indicating texture and detail information. SCD evaluates the image fusion quality by comparing the structure, content, and distortion levels between the original images. VIF is used to assess the ability of the image to retain original information during transmission or processing. Q^AB/F^ reflects the visual information quality in the fused image. All these metrics are positively correlated with image fusion quality, meaning that the higher the value of the evaluation metric, the better the fusion quality.

### Comparison experiment

In order to verify the validity of the Long-Range Correlation-Guided Dual-Encoder Fusion Network for Medical Images, 9 comparative experiments are carried out. In Section “CT lung window image and PET image group”, 10 methods are qualitatively evaluated for 200 pairs of CT lung window images and PET images, and 8 evaluation metrics are quantitatively evaluated for the fused images. In Section “CT mediastinal window image and PET image group”, 10 methods are qualitatively evaluated for 200 pairs of CT mediastinal window images and PET images, and 8 evaluation metrics are quantitatively evaluated for the fused images. In Section “MRI brain image and PET image group”, 10 methods are qualitatively evaluated for 100 pairs of MRI brain images and PET images, and 8 evaluation metrics are quantitatively evaluated for the fused images.In Section “Ablation experiment 4: ablation of pooling size”, ablation experiments are performed on the pooling size.

#### CT lung window image and PET image group

**Figure 4:**
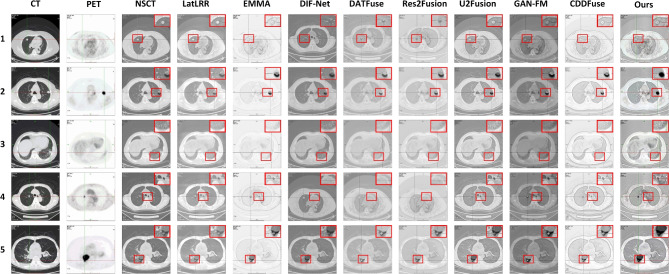
The comparison experiments fusion results of CT lung window images and PET images. Method 1: NSCT; Method 2: LatLRR; Method 3: EMMA; Method 4: DIF-Net; Method 5: DATFuse; Method 6: Res2Fusion; Method 7: U2Fusion; Method 8: GAN-FM; Method 9: CDDFuse; Method 10: Ours.

**Figure 5:**
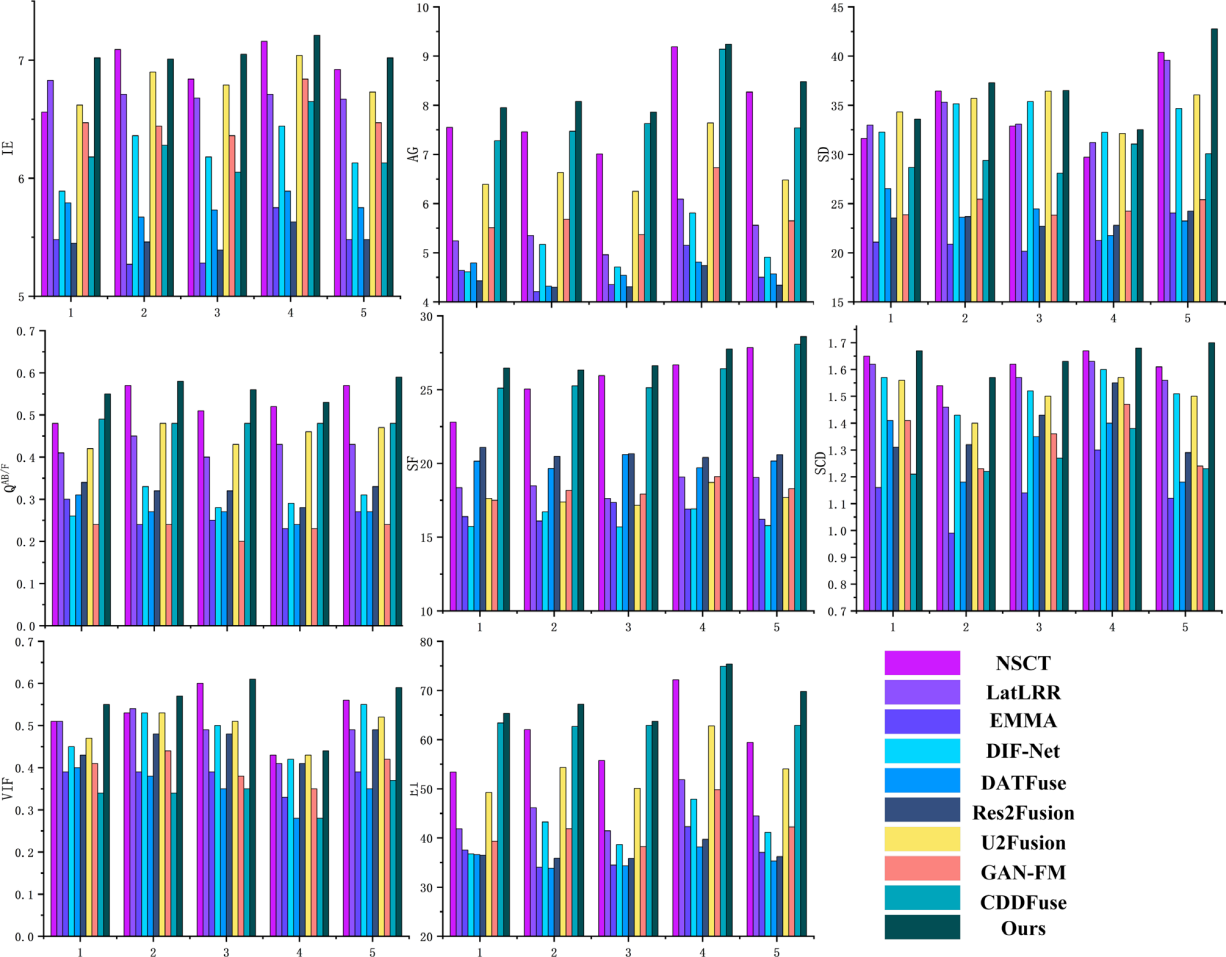
Histogram of the evaluation metrics of CT lung window fusion images.

In this section, 200 pairs of CT lung window images and PET images are divided into 5 groups, with 40 pairs of CT lung window images and PET images in each group, and 10 comparison methods are used for comparison. 5 groups of visualization fusion results are selected, and the fusion results are shown in Fig. 4. In Fig. 4, columns 3 and 4 are results of the first set, and columns 5 through 12 are results of the second set. Table 1 shows the average results of evaluation metrics for each group of fused images. Among them, the best evaluation metric value is represented in red, and the second-best evaluation metrics are represented in blue. The histogram of the average evaluation metrics for the fused images is shown in Fig. 5.

As shown in Fig. 4, Methods 2, 3, 6, and 9 generate relatively clear fused images, but Methods 1 and 9 suffer from overly high brightness, weak lesion information, and unclear textures, making it difficult to accurately identify the detailed lung bronchial structures in the CT source images. Methods 3 and 7 accurately locate the lesion areas, but their contrast is low, resulting in the lesion is not prominent. Among them, Method 3 is severely exposed, which impairs the observation of details. Method 7 enables better retain the edge and texture information of CT source images, but its lesion information is not obvious. Method 1 generates fusion images with clear lesions but its ability to retain gradient information is poor, which makes it difficult to recognize edge and bone information in CT source images. The fusion images obtained by methods 4 and 8 are generally dark with poor visual effects. Moreover, the lesion information perception ability of method 8 is weak, which makes it difficult to locate the lesion area effectively. Methods 5 and 7 result in fusion images that are blurry with high brightness, resulting in the contrast between the region information and the background region information is not obvious. In contrast, the method proposed in this paper not only retains the edge and contour information from the CT source images effectively but also enhances the lesion information from the PET source images.

**Table 1:** The comparison experiments evaluation metrics of CT lung window images and PET images (Bold: best; Bolditalic: second best).

Image	Methods	IE	AG	SD	SF	SCD	VIF	${\textrm{Q}^{\textrm{AB}/\textrm{F}}}$	EI
**1**	NSCT	6.56	***7.55***	31.61	22.79	***1.65***	***0.51***	0.48	53.41
	LatLRR	***6.83***	5.24	32.98	18.36	1.62	0.51	0.41	41.85
	EMMA	5.48	4.64	21.08	16.39	1.16	0.39	0.30	37.52
	DIF-Net	5.89	4.61	32.27	15.73	1.57	0.45	0.26	36.76
	DATFuse	5.79	4.79	26.52	20.15	1.41	0.40	0.31	36.60
	Res2Fusion	5.45	4.43	23.54	21.08	1.31	0.43	0.34	36.46
	U2Fusion	6.62	6.39	**33.82**	17.62	1.56	0.47	0.42	49.27
	GAN-FM	6.47	5.51	23.87	17.50	1.41	0.41	0.24	39.32
	CDDFuse	6.18	7.28	28.68	***25.10***	1.21	0.34	***0.49***	***63.38***
	Ours	**7.02**	**7.95**	***33.60***	**26.47**	**1.67**	**0.55**	**0.55**	**65.32**
**2**	NSCT	**7.09**	7.46	***36.44***	25.05	***1.54***	0.53	***0.57***	62.03
	LatLRR	6.71	5.35	35.32	18.48	1.46	***0.54***	0.45	46.13
	EMMA	5.27	4.21	20.87	16.10	0.99	0.39	0.24	34.04
	DIF-Net	6.36	5.17	35.14	16.71	1.43	0.53	0.33	43.24
	DATFuse	5.67	4.32	23.61	19.65	1.18	0.38	0.27	33.82
	Res2Fusion	5.46	4.30	23.68	20.47	1.32	0.48	0.32	35.87
	U2Fusion	6.90	6.63	35.69	17.38	1.40	0.53	0.48	54.35
	GAN-FM	6.44	5.68	25.45	18.16	1.23	0.44	0.24	41.87
	CDDFuse	6.28	***7.47***	29.38	***25.26***	1.22	0.34	0.48	***62.66***
	Ours	***7.01***	**8.08**	**37.28**	**26.33**	**1.57**	**0.57**	**0.58**	**67.19**
**3**	NSCT	***6.84***	7.01	32.87	***25.95***	***1.62***	***0.60***	***0.51***	55.73
	LatLRR	6.68	4.96	33.09	17.62	1.57	0.49	0.40	41.45
	EMMA	5.28	4.35	20.15	17.35	1.14	0.39	0.25	34.47
	DIF-Net	6.18	4.71	35.39	15.70	1.52	0.50	0.28	38.67
	DATFuse	5.73	4.54	24.47	20.60	1.35	0.35	0.27	34.36
	Res2Fusion	5.39	4.31	22.70	20.66	1.43	0.48	0.32	35.83
	U2Fusion	6.79	6.25	***36.43***	17.16	1.50	0.51	0.43	50.10
	GAN-FM	6.36	5.37	23.82	17.92	1.36	0.38	0.20	38.23
	CDDFuse	6.05	***7.63***	28.08	25.13	1.27	0.35	0.48	***62.90***
	Ours	**7.05**	**7.86**	**36.50**	**26.62**	**1.63**	**0.61**	**0.56**	**63.72**
4	NSCT	***7.16***	***9.19***	29.72	***26.68***	***1.67***	***0.43***	***0.52***	72.14
	LatLRR	6.71	6.09	31.20	19.07	1.63	0.41	0.43	51.86
	EMMA	5.75	5.15	21.25	16.91	1.30	0.33	0.23	42.30
	DIF-Net	6.44	5.81	***32.26***	16.92	1.60	0.42	0.29	47.86
	DATFuse	5.89	4.81	21.75	19.70	1.40	0.28	0.24	38.14
	Res2Fusion	5.63	4.74	22.79	20.40	1.55	0.41	0.28	39.71
	U2Fusion	7.04	7.64	32.12	18.71	1.57	***0.43***	0.46	62.77
	GAN-FM	6.84	6.73	24.22	19.10	1.47	0.35	0.23	49.81
	CDDFuse	6.65	9.14	31.06	26.42	1.38	0.28	0.48	***74.89***
	Ours	**7.21**	**9.24**	**32.51**	**27.75**	**1.68**	**0.44**	**0.53**	**75.39**
5	NSCT	***6.92***	***8.27***	***40.38***	27.85	***1.61***	***0.56***	***0.57***	59.41
	LatLRR	6.67	5.56	39.57	19.04	1.56	0.49	0.43	44.47
	EMMA	5.48	4.50	24.05	16.21	1.12	0.39	0.27	37.07
	DIF-Net	6.13	4.91	34.66	15.78	1.51	0.55	0.31	41.14
	DATFuse	5.75	4.57	23.23	20.16	1.18	0.35	0.27	35.31
	Res2Fusion	5.48	4.34	24.23	20.59	1.29	0.49	0.33	36.20
	U2Fusion	6.73	6.48	36.05	17.68	1.50	0.52	0.47	54.03
	GAN-FM	6.47	5.65	25.39	18.29	1.24	0.42	0.24	42.24
	CDDFuse	6.13	7.54	30.08	***28.07***	1.23	0.37	0.48	***62.89***
	Ours	**7.02**	**8.48**	**42.78**	**28.60**	**1.70**	**0.59**	**0.59**	**69.78**

As shown in Table 1 and Fig. 5, there is little difference between the proposed method and NSCT in IE and SD. Compared with the highest value of the comparison method, the proposes method improves by an average increase of 4.55%, 5.64% and 4.49%, respectively, and compared with the lowest value of the comparison method, the proposed method improves by an average of 4.55%, 5.64%, and 4.49%, respectively. Therefore, the proposed method in this paper shows better performance in fusing CT lung window images and PET images.

#### CT mediastinal window image and PET image group

**Figure 6:**
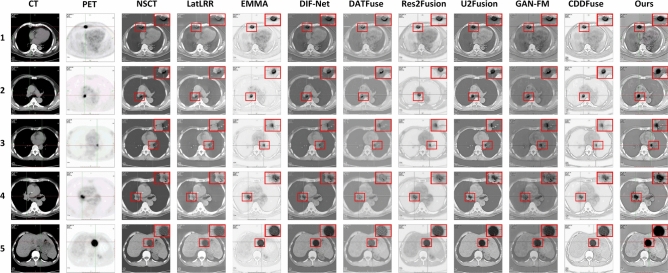
The comparison experiments fusion results of CT mediastinal window images and PET images. Method 1: NSCT; Method 2: LatLRR; Method 3: EMMA; Method 4: DIF-Net; Method 5: DATFuse; Method 6: Res2Fusion; Method 7: U2Fusion; Method 8: GAN-FM; Method 9: CDDFuse; Method 10: Ours.

**Figure 7:**
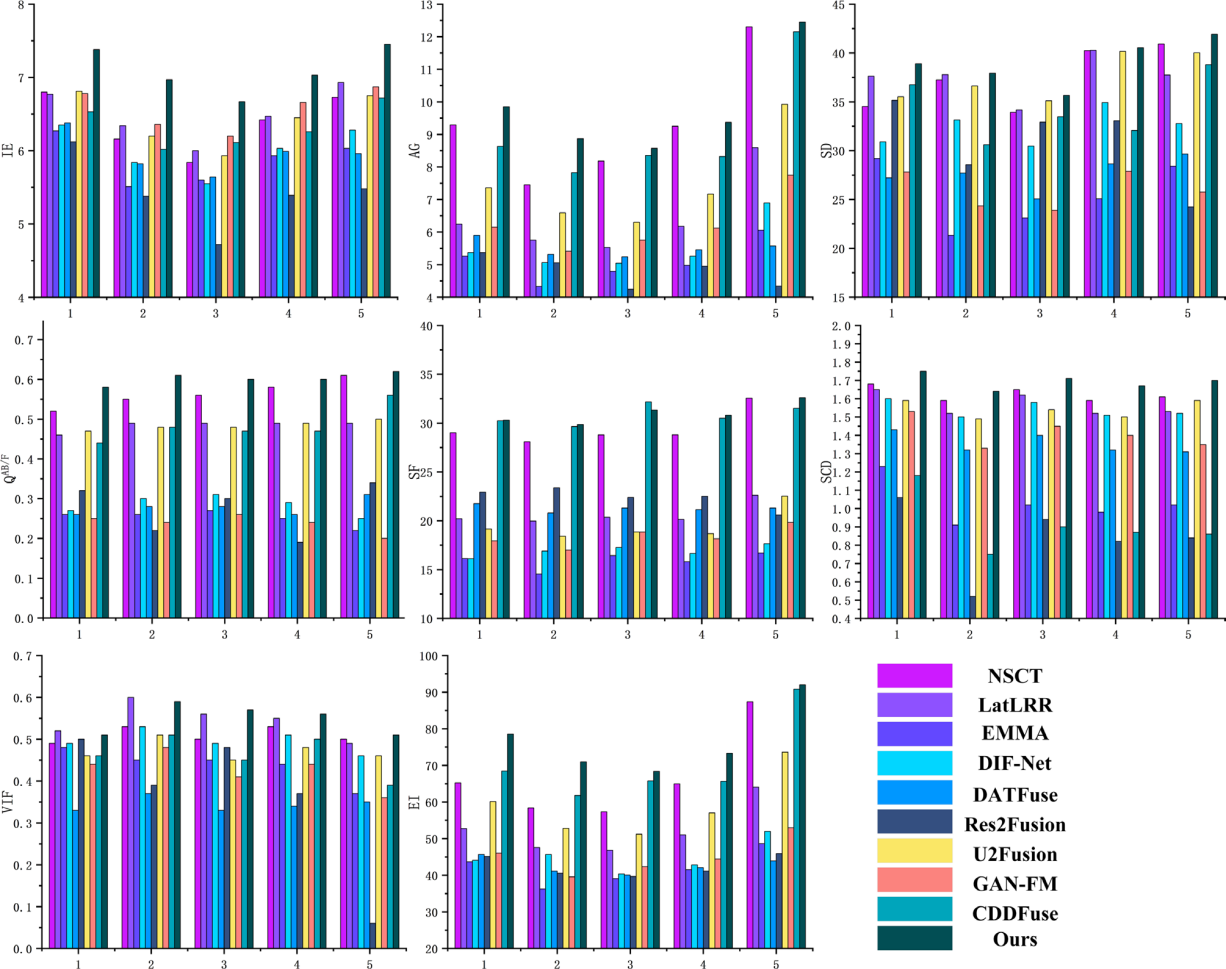
Histogram of the evaluation metrics of CT mediastinal window fusion images.

In this section, 200 pairs of CT lung window images and PET images are divided into 5 groups, with 40 pairs of CT mediastinal window images and PET images in each group, and 10 comparison methods are used for comparison. 5 groups of visualization fusion results are selected, and the fusion results are shown in Fig. 6. In Fig. 6, columns 3 and 4 are results of the first set, and columns 5 through 12 are results of the second set. Table 2 shows the average results of evaluation metrics for each group of fused images. Among them, the best evaluation metric value is represented in red, and the second-best evaluation metric is represented in blue. The histogram of the average evaluation metrics for the fused images is shown in Fig. 7.

**Table 2:** The comparison experiments evaluation metrics of CT mediastinal window images and PET images (Bold: best; Bolditalic: second best).

Image	Methods	IE	AG	SD	SF	SCD	VIF	${\textrm{Q}^{\textrm{AB}/\textrm{F}}}$	EI
1	NSCT	6.80	***9.29***	34.52	29.01	***1.68***	0.49	***0.52***	65.26
	LatLRR	6.77	6.24	***37.63***	20.21	1.65	**0.52**	0.46	52.72
	EMMA	6.27	5.26	29.19	16.12	1.23	0.48	0.26	43.66
	DIF-Net	6.35	5.37	30.90	16.11	1.60	0.49	0.27	44.09
	DATFuse	6.38	5.90	27.22	21.75	1.43	0.33	0.26	45.66
	Res2Fusion	6.12	5.37	35.15	22.94	1.06	0.50	0.32	45.15
	U2Fusion	***6.81***	7.36	35.52	19.15	1.59	0.46	0.47	60.12
	GAN-FM	6.78	6.15	27.80	17.94	1.53	0.44	0.25	46.04
	CDDFuse	6.53	8.63	***36.76***	***30.24***	1.18	0.46	0.44	***68.47***
	Ours	**7.38**	**9.85**	**38.91**	**30.30**	**1.75**	***0.51***	**0.58**	**78.53**
2	NSCT	6.16	7.45	37.25	28.09	***1.59***	0.53	***0.55***	58.35
	LatLRR	6.34	5.75	***37.78***	19.97	1.52	**0.60**	0.49	47.59
	EMMA	5.51	4.33	21.33	14.55	0.91	0.45	0.26	36.22
	DIF-Net	5.84	5.06	33.14	16.90	1.50	0.53	0.30	45.66
	DATFuse	5.82	5.31	27.69	20.80	1.32	0.37	0.28	41.11
	Res2Fusion	5.38	5.05	28.55	23.39	0.52	0.39	0.22	40.56
	U2Fusion	6.20	6.59	36.63	18.42	1.49	0.51	0.48	52.78
	GAN-FM	***6.36***	5.41	24.34	16.99	1.33	0.48	0.24	39.57
	CDDFuse	6.02	***7.82***	30.61	***29.65***	0.75	0.51	0.48	***61.79***
	Ours	**6.97**	**8.87**	**37.93**	**29.86**	**1.64**	***0.59***	**0.61**	**70.99**
3	NSCT	5.84	8.18	33.91	28.79	***1.65***	0.50	***0.56***	57.30
	LatLRR	6.00	5.53	34.18	20.36	1.62	***0.56***	0.49	46.81
	EMMA	5.60	4.79	23.11	16.44	1.02	0.45	0.27	39.08
	DIF-Net	5.55	5.04	30.47	17.27	1.58	0.49	0.31	40.39
	DATFuse	5.64	5.24	25.05	21.30	1.40	0.33	0.28	40.04
	Res2Fusion	4.72	4.24	32.92	22.39	0.94	0.48	0.30	39.66
	U2Fusion	5.93	6.30	***35.11***	18.85	1.54	0.45	0.48	51.21
	GAN-FM	***6.20***	5.75	23.89	18.84	1.45	0.41	0.26	42.34
	CDDFuse	6.11	***8.35***	33.46	***31.18***	0.90	0.45	0.47	***65.74***
	Ours	**6.67**	**8.57**	**35.65**	**31.35**	**1.71**	**0.57**	**0.60**	**68.39**
4	NSCT	6.42	***9.25***	40.25	28.80	***1.59***	0.53	***0.58***	64.98
	LatLRR	6.47	6.18	***40.28***	20.13	1.52	***0.55***	0.49	51.04
	EMMA	5.93	4.98	25.06	15.80	0.98	0.44	0.25	41.54
	DIF-Net	6.03	5.26	34.93	16.65	1.51	0.51	0.29	42.85
	DATFuse	5.99	5.45	28.63	21.14	1.32	0.34	0.26	42.06
	Res2Fusion	5.39	4.95	33.06	22.51	0.82	0.37	0.19	41.14
	U2Fusion	6.45	7.16	40.16	18.68	1.50	0.48	0.49	57.02
	GAN-FM	***6.66***	6.12	27.89	18.16	1.40	0.44	0.24	44.40
	CDDFuse	6.26	***8.32***	32.07	***30.51***	0.87	0.50	0.47	***65.61***
	Ours	**7.03**	**9.37**	**40.54**	**30.81**	**1.67**	**0.56**	**0.60**	**73.33**
5	NSCT	6.73	***12.30***	***40.91***	***32.56***	***1.61***	***0.50***	***0.61***	87.40
	LatLRR	***6.93***	8.59	37.75	22.61	1.53	0.49	0.49	64.10
	EMMA	6.03	6.06	28.41	16.70	1.02	0.37	0.22	48.67
	DIF-Net	6.28	6.89	32.78	17.65	1.52	0.46	0.25	51.99
	DATFuse	5.96	5.57	29.66	21.30	1.31	0.35	0.31	43.93
	Res2Fusion	5.48	4.34	24.23	20.59	0.84	0.06	0.34	45.90
	U2Fusion	6.75	9.92	40.01	22.53	1.59	0.46	0.50	73.62
	GAN-FM	6.87	7.75	25.77	19.83	1.35	0.36	0.20	52.97
	CDDFuse	6.72	12.15	38.80	31.51	0.86	0.39	0.56	***90.85***
	Ours	**7.45**	**12.45**	**41.92**	**32.61**	**1.70**	**0.51**	**0.62**	**92.03**

As shown in Fig. 6, Methods 1, 4, and 10 are capable of generating clear fusion images and accurately locating the lesion area. However, the images from Methods 1 ,4 and 7 exhibit low overall contrast and blurred details, with a lack of clear edge information. Method 2 generates fused images with a clear lesion area and high contrast. However, the exposure is overly high, the edge and texture information cannot be clearly and accurately identified. Method 8 generates fused image that lacks prominent lesion information in the lesion area. The lesion information of fusion images generated by methods 3 and 6 is weak and unclear. The fusion images generated by method 5 and method 9 are blurred, the visual effect is poor, and the contrast between the lesion information and the background information is not obvious. Method 10 generates fused images that are clearer, and the contrast between the lesion area and the background area is obvious, which enables effectively highlights the lesion area. It not only retains the bone and edge contour information of the CT source images, but also highlights the lesion information of the PET source image.

As shown in Table 2 and Fig. 7, there is little difference between the proposed method and NSCT in SCD, and CDDFuse in EI. However, our method performs better in AG, Q^AB/F^, and EI, with average increases of 4.47%, 6.74%, and 8.74% over the highest values of the comparison methods, and average increases of 112.41%, 168.75%, and 87.85% over the lowest values. Therefore, our method achieves clear edge textures and lesion regions, resulting in good visual effects in the fused images.

####  MRI brain image and PET image group

**Figure 8:**
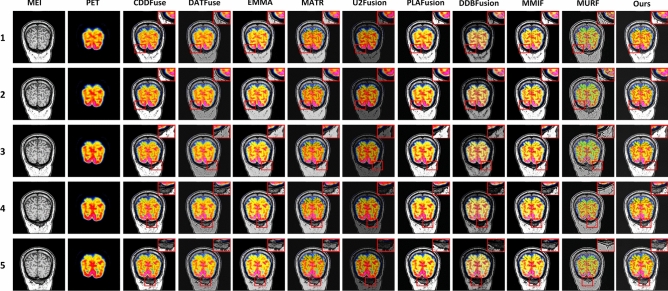
The comparison experiments fusion results of MRI brain images and PET images. Method 1: CDDFuse; Method 2: DATFuse; Method 3: EMMA; Method 4: MATR; Method 5: U2Fusion; Method 6: PLAFusion; Method 7: DDBFusion; Method 8: MMIF; Method 9: MURF; Method 10: Ours.

**Figure 9:**
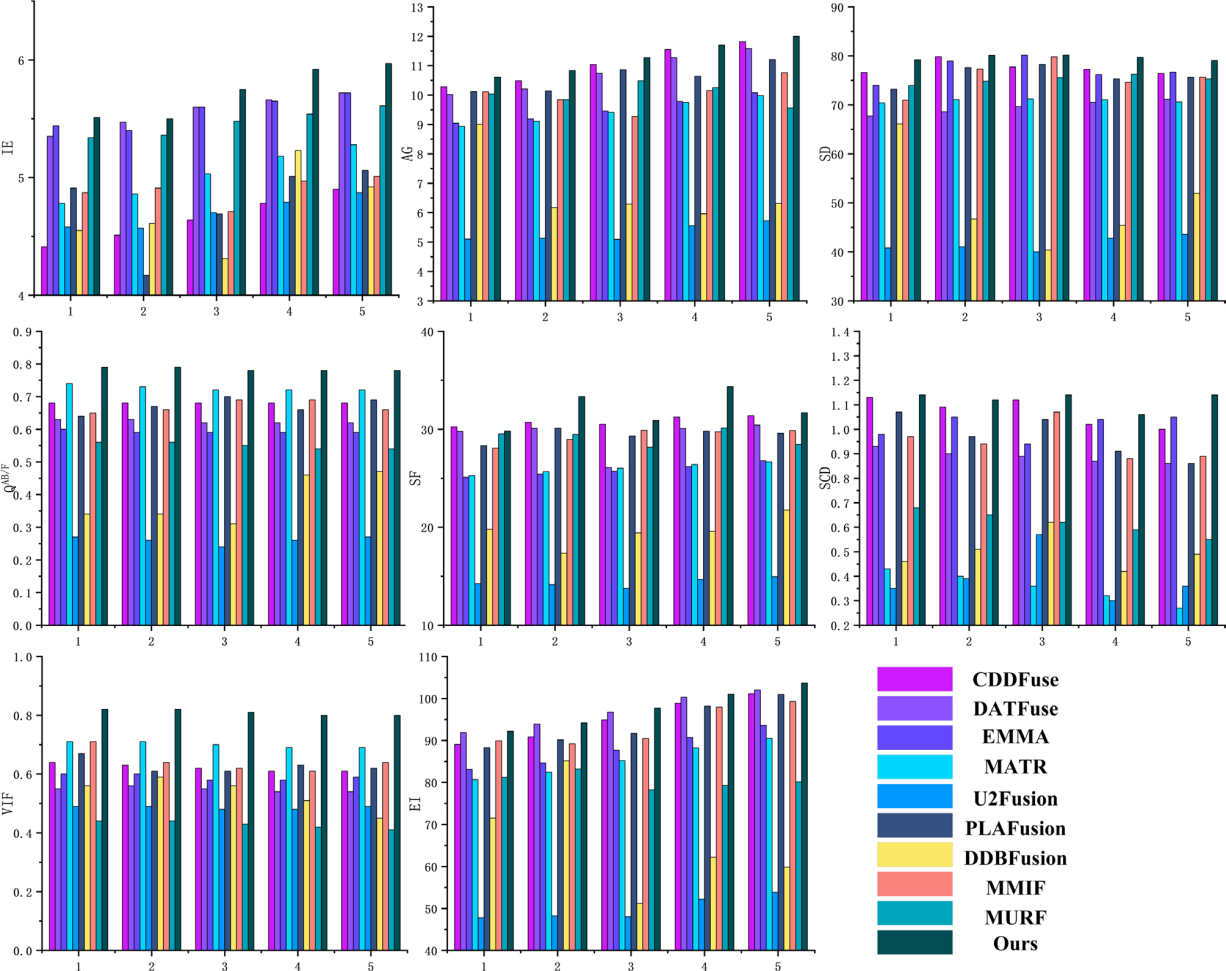
Histogram of the evaluation metrics of MRI brain images and PET images.

In this section, 100 pairs of MRI brain images and PET images are divided into 5 groups, with 20 pairs of MRI brain images and PET images in each group, and 10 comparison methods are used for comparison. 5 groups of visualization fusion results were selected, and the fusion results are shown in Fig. 8. Table 3 shows the average results of evaluation metrics for each group of fused images. Among them, the best evaluation metric value is represented in red, and the second-best evaluation metric is represented in blue. The histogram of the average evaluation metrics for the fused images is shown in Fig. 9.

As shown in Fig. 8, Methods 2, 3, 4, and 9 better retain the color features of the PET images, but the structural details of the MRI are insufficient, the tissue level of the brain is not obvious, and the contrast is low, which leads to the unclear distinction between the lesion area and the surrounding structure. Methods 1, 6 and Method 10 not only retain the color information of PET, but also well preserve the structural details of MRI. However, method 10 has higher contrast and clear edges, and has a clear sense of brain hierarchy and rich structural information.

**Table 3:** The comparison experiments evaluation metrics of MRI brain images and PET images (Bold: best; Bolditalic: second best).

Image	Methods	IE	AG	SD	SF	SCD	VIF	${\textrm{Q}^{\textrm{AB}/\textrm{F}}}$	EI
1	CDDF	4.41	***10.28***	***76.61***	**30.25**	***1.13***	0.64	0.68	89.06
	DATFuse	5.35	10.02	67.73	29.79	0.93	0.55	0.63	***91.90***
	EMMA	***5.44***	9.04	74.02	25.10	0.98	0.60	0.60	83.11
	MATR	4.78	8.93	70.41	25.27	0.43	***0.71***	***0.74***	80.69
	U2Fusion	4.58	5.10	40.79	14.24	0.35	0.49	0.27	47.79
	PLAFusion	4.91	10.12	73.21	28.32	1.07	0.67	0.64	88.26
	DDBFusion	4.55	9.01	66.13	19.78	0.46	0.56	0.34	71.52
	MMIF	4.87	10.11	70.94	28.07	0.97	***0.71***	0.65	89.94
	MURF	5.34	10.04	73.94	29.52	0.68	0.44	0.56	81.21
	Ours	**5.51**	**10.61**	**79.20**	***29.82***	**1.14**	**0.82**	**0.79**	**92.21**
2	CDDF	4.51	***10.49***	***79.82***	***30.71***	***1.09***	0.63	0.68	90.85
	DATFuse	***5.47***	10.21	68.60	30.10	0.90	0.56	0.63	***93.91***
	EMMA	5.40	9.19	78.95	25.43	1.05	0.60	0.59	84.66
	MATR	4.86	9.11	71.07	25.66	0.40	***0.71***	***0.73***	82.46
	U2Fusion	4.57	5.13	41.01	14.14	0.39	0.49	0.26	48.21
	PLAFusion	4.17	10.14	77.62	30.11	0.97	0.61	0.67	90.16
	DDBFusion	4.61	6.17	46.72	17.34	0.51	0.59	0.34	85.13
	MMIF	4.91	9.84	77.31	28.97	0.94	0.64	0.66	89.19
	MURF	5.36	9.84	74.85	29.49	0.65	0.44	0.56	83.19
	Ours	**5.50**	**10.83**	**80.16**	**33.34**	**1.12**	**0.82**	**0.79**	**94.21**
3	CDDF	4.78	***11.04***	77.24	***30.51***	***1.12***	0.61	0.68	94.91
	DATFuse	***5.60***	10.74	70.54	30.08	0.87	0.54	0.62	***96.76***
	EMMA	***5.60***	9.46	***80.17***	25.71	0.94	0.58	0.59	87.70
	MATR	5.03	9.41	71.22	26.03	0.36	***0.70***	***0.72***	85.18
	U2Fusion	4.70	5.09	39.97	13.76	0.57	0.48	0.24	48.03
	PLAFusion	4.69	10.86	78.25	29.31	1.04	0.61	0.70	91.73
	DDBFusion	4.31	6.29	40.39	19.43	0.62	0.56	0.31	51.14
	MMIF	4.71	9.27	79.81	29.91	1.07	0.62	0.69	90.47
	MURF	5.48	10.49	75.56	28.16	0.62	0.43	0.55	78.25
	Ours	**5.75**	**11.27**	**80.20**	**30.89**	**1.14**	**0.81**	**0.78**	**97.73**
4	CDDF	4.78	***11.55***	***77.24***	***31.25***	1.00	0.61	0.68	98.87
	DATFuse	***5.66***	11.58	71.17	30.43	0.86	0.54	0.62	***100.30***
	EMMA	5.65	9.78	76.15	26.17	***1.04***	0.58	0.59	90.73
	MATR	5.18	9.75	71.04	26.40	0.32	***0.69***	***0.72***	88.21
	U2Fusion	4.79	5.55	42.77	14.67	0.30	0.48	0.26	52.19
	PLAFusion	5.01	10.64	75.31	29.81	0.91	0.63	0.66	98.17
	DDBFusion	5.23	5.96	45.38	19.57	0.42	0.51	0.46	62.19
	MMIF	4.97	10.15	74.61	29.74	0.88	0.61	0.69	97.96
	MURF	5.54	10.25	76.25	30.12	0.59	0.42	0.54	79.27
	Ours	**5.92**	**11.70**	**79.68**	**34.34**	**1.06**	**0.80**	**0.78**	**101.06**
5	CDDF	4.90	***11.82***	76.41	***31.38***	1.00	0.61	0.68	101.16
	DATFuse	***5.72***	11.58	71.17	30.43	0.86	0.54	0.62	***102.05***
	EMMA	***5.72***	10.08	***76.68***	26.81	***1.05***	0.59	0.59	93.60
	MATR	5.28	9.98	70.62	26.67	0.27	***0.69***	***0.72***	90.48
	U2Fusion	4.87	5.72	43.57	14.95	0.36	0.49	0.27	53.88
	PLAFusion	5.06	11.21	75.64	29.59	0.86	0.62	0.69	100.98
	DDBFusion	4.92	6.31	51.98	21.75	0.49	0.45	0.47	59.83
	MMIF	5.01	10.76	75.67	29.87	0.89	0.64	0.66	99.29
	MURF	5.61	9.56	75.31	28.46	0.55	0.41	0.54	80.15
	Ours	**5.97**	**12.00**	**79.06**	**31.69**	**1.14**	**0.80**	**0.78**	**103.67**

As shown in Table 3 and Fig. 9, there is little difference between the proposes method and CDDF in AG, and DATFuse in IE and EI. However, our method performs better in SF, VIF, and Q^AB/F^, with average increases of 3.88%, 15.71%, and 7.99% over the highest values of the comparison methods, and average increases of 123.08%, 89.25%, and 201.54% over the lowest values. Therefore, this paper gains a clear edge textures and lesion regions, resulting in good visual effects in the fused images.

### Ablation experiment

In order to verify the effectiveness of each module of the Long-Range Correlation-Guided Dual-Encoder Fusion Network for Medical lmages, 3 CT lung window images and PET images, 3 CT mediastinal window images and PET images, and 3 MRI brain images and PET images are selected for ablation experiments to verify the effectiveness of the proposed method. Exp1: Remove all the modules design in this paper, and use the basic encoder–decoder network (Base) for feature extraction, and adopts the direct addition fusion strategy for fusion. Exp2: Dual-encoder Single-decoder network architecture is used to verify the effectiveness of enhancing fine-grained features from different modals. Exp3: Based on Exp2, CMFEM is added to validate its effectiveness. Exp4: Based on Exp3, only the last stage of CMFEM - LCFM is used for feature fusion. Exp5: Long-Range Correlation-Guided Dual-Encoder Fusion Network for Medical lmages. The details are shown in Table 4.

**Table 4:** Ablation experiment module setup, where $\checkmark$ means the module is included and $\times$ means the module is not included.

Method	Base	Dense aggregate dual-encoder	CMFEM	LCFM (1)	LCFM
Exp1	$\checkmark$	$\times$	$\times$	$\times$	$\times$
Exp2	$\checkmark$	$\checkmark$	$\times$	$\times$	$\times$
Exp3	$\checkmark$	$\checkmark$	$\checkmark$	$\times$	$\times$
Exp4	$\checkmark$	$\checkmark$	$\checkmark$	$\checkmark$	$\times$
Exp5	$\checkmark$	$\checkmark$	$\checkmark$	$\times$	$\checkmark$

#### Ablation experiment 1: CT lung window image and PET image

**Figure 10:**
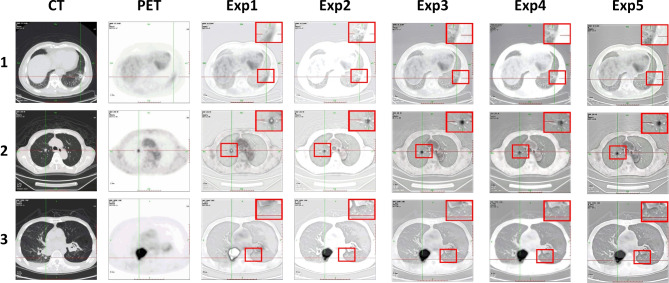
The ablation experiments fusion results of CT lung window images and PET images.

In Fig. 10, the fusion image is generated by Exp1 retains some edge information from the CT source image and lesion information in the PET source image. However, because the dense aggregate encoder, CMFEM and LCFM modules are not included, the direct addition fusion strategy is adopted, which leads to the lesion area is not significant enough, and the contrast between the lesion and the background is low. In contrast, the fusion images from Exp2 show improve edge and texture information, indicating that the dense Aggregate dual-encoder is more effective at preserving the structural details. However, due to the lack of CMFEM and LCFM modules, the intensity distribution of the image is uneven, resulting in unclear lesion information. In the fusion image of Exp3, the lesion area is more prominent, and the contrast between the lesion and the background is significantly improved, which indicates the effectiveness of CMFEM module in capturing image intensity distribution. However, due to the lack of LCFM module, the edge and intensity information extracted by the fusion strategy of direct addition is insufficient, resulting in high overall image brightness. Compared with Exp3, Exp4 fusion image improves brightness, but there are still artifacts around the lesion, and the edge and detail information are poor. Exp5, the method proposed in this paper generates a fusion image that effectively preserves edge and texture information, with the lesion area clearly visible.

**Table 5:** The ablation experiments evaluation metrics values of CT lung window images and PET images (Bold: best; Bolditalic: second best).

Image	Methods	IE	AG	SD	SF	SCD	VIF	${\textrm{Q}^{\textrm{AB}/\textrm{F}}}$	EI
1	Exp1	4.9	5.67	27.44	24.68	1.34	0.35	0.35	43.10
	Exp2	5	5.77	27.82	26.14	1.50	0.38	0.37	44.97
	Exp3	***6.73***	***7.36***	34.22	***26.47***	1.57	***0.58***	***0.46***	49.11
	Exp4	6.37	6.26	***35.07***	24.78	***1.61***	0.56	0.40	***56.45***
	Exp5	**7.05**	**7.86**	**36.50**	**26.62**	**1.63**	**0.61**	**0.56**	**63.72**
2	Exp1	5.24	6.90	27.98	23.60	1.37	0.33	0.31	55.22
	Exp2	5.95	7.13	28.66	27.26	1.62	0.36	0.36	55.74
	Exp3	***6.91***	***8.85***	***31.17***	***27.41***	1.60	0.39	***0.42***	60.26
	Exp4	6.89	7.78	31.14	24.51	***1.65***	***0.41***	0.38	***68.57***
	Exp5	**7.21**	**9.24**	**32.51**	**27.75**	**1.68**	**0.60**	**0.53**	**75.39**
3	Exp1	5.05	6.11	26.95	23.11	1.04	0.32	0.35	49.78
	Exp2	5.45	6.56	34.66	27.32	1.56	0.44	0.43	52.66
	Exp3	***6.75***	***7.89***	38.22	***27.93***	1.60	0.48	***0.46***	52.86
	Exp4	6.34	6.44	***41.95***	23.53	***1.67***	***0.53***	0.42	***61.3***
	Exp5	**7.02**	**8.48**	**42.78**	**28.6**	**1.70**	**0.59**	**0.59**	**69.78**

**Figure 11:**
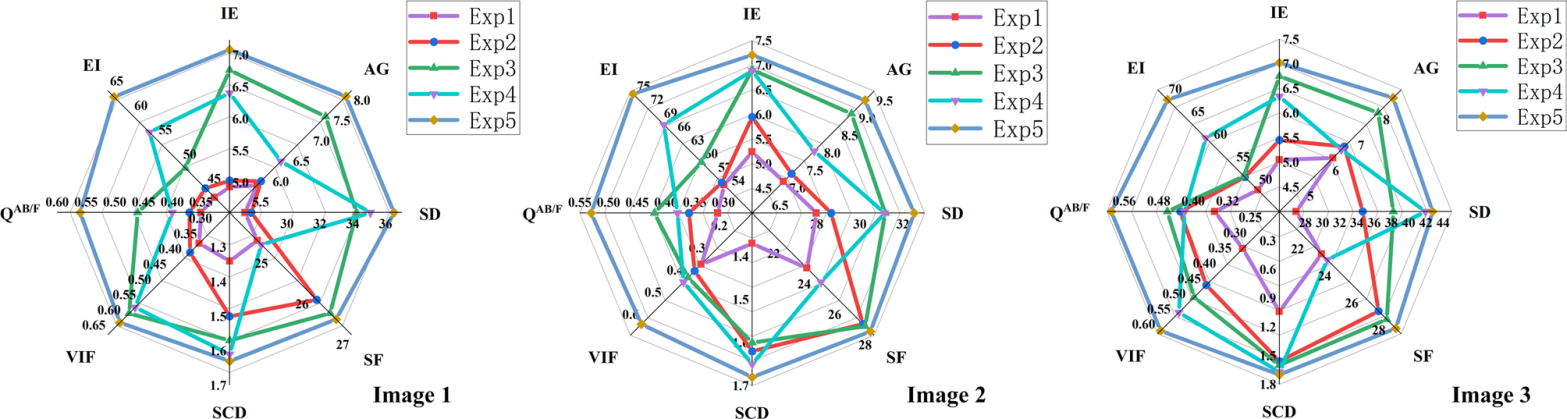
The ablation experiments evaluation metrics coefficient radar maps of CT lung window images and PET images.

As shown in Table 5 and Fig. 11, The evaluation metrics value of Exp1 is the lowest. Compared with Exp1, the values of various evaluation metrics of Exp3 and Exp4 have little difference from the proposed method, but they are all lower than the proposed method. For example, the SCD of Exp4 is second only to the proposed method. The above results reflect that the fusion images obtained by the proposed method have certain advantages in both subjective and objective evaluation.

**Figure 12:**
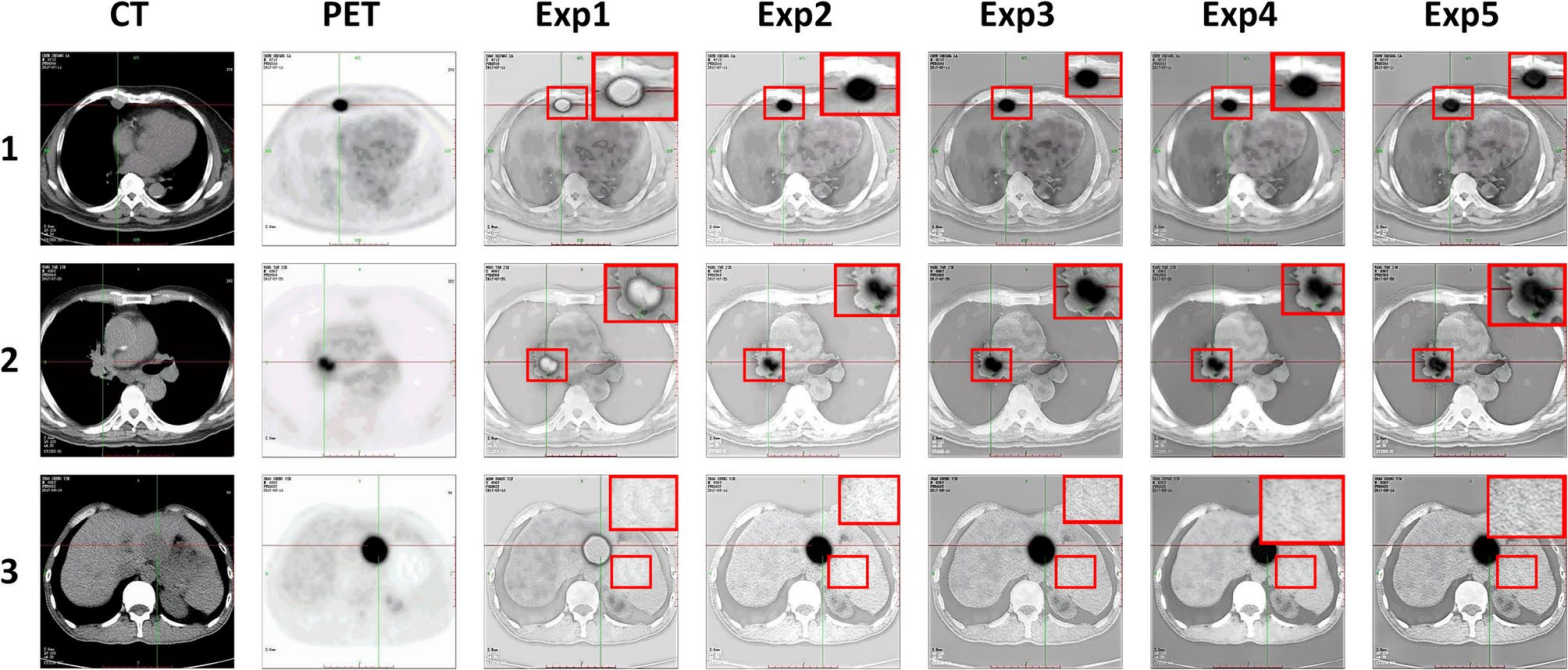
The ablation experiments fusion results of CT mediastinal window images and PET images.

**Table 6:** The ablation experiments evaluation metrics values of CT lung window images and PET images (Bold: best; Bolditalic: second best).

Image	Methods	IE	AG	SD	SF	SCD	VIF	${\textrm{Q}^{\textrm{AB}/\textrm{F}}}$	EI
**1**	Exp1	6.7	7.12	32.47	23.46	1.23	0.4	0.32	57.65
	Exp2	6.71	8.76	36.81	29.57	1.68	***0.48***	***0.46***	68.94
	Exp3	***7.21***	***9.69***	35.45	***29.8***	1.6	0.42	0.44	***69.84***
	Exp4	7.08	7.69	***37.08***	24.88	***1.69***	0.48	0.38	58.85
	Exp5	**7.38**	**9.85**	**38.91**	**30.3**	**1.75**	**0.51**	**0.58**	**78.53**
**2**	Exp1	6.44	7.2	29.68	24.74	1.21	0.39	0.4	52.19
	Exp2	6.52	7.99	33.07	***28.34***	1.56	0.42	***0.49***	63.1
	Exp3	***6.82***	***8.91***	36.7	27.97	1.56	0.47	0.42	***65.1***
	Exp4	6.7	7.55	***39.8***	25.47	***1.61***	0.49	0.41	54.39
	Exp5	**7.03**	**9.17**	**40.54**	**28.81**	**1.67**	**0.55**	**0.6**	**73.33**
**3**	Exp1	6.48	8.95	28.58	24.26	0.96	0.35	0.36	61.01
	Exp2	6.54	10.65	38.64	30.7	1.6	0.42	***0.44***	77.47
	Exp3	***7.1***	***12.34***	37.79	***31.99***	1.56	***0.43***	0.39	***78.91***
	Exp4	7.12	9.5	***41.33***	26.82	***1.65***	0.41	0.41	64.13
	Exp5	**7.45**	**12.45**	**41.92**	**32.61**	**1.7**	**0.45**	**0.58**	**92.03**

#### Ablation experiment 2: CT mediastinal window image and PET image

The results of CT mediastinal window images and PET images ablation experiments are consistent with the fusion results of CT lung window images and PET images ablation experiments. As shown in Fig. 12, the overall effect of Exp1 fusion image is poor, the lesion area is not prominent, and the contrast is low. Exp2 retains more edge and texture information, and CT structure information is clearer, but the brightness distribution is uneven, resulting in local brightness distortion. In the fusion image of Exp3, the lesion area is more prominent, and the contrast between the lesion and the background is enhanced, but the overall brightness of the image is high, and the detail performance is still insufficient. Exp4 not only makes the lesion information more prominent, but also improves the brightness distribution, and CT structure information is retained, but there are still artifacts and blurring phenomena in the detailed areas. Exp5 is the method proposed in this paper. The fusion images are clear in edges and details, the lesion area is prominent and the overall visual quality is better.

**Figure 13:**
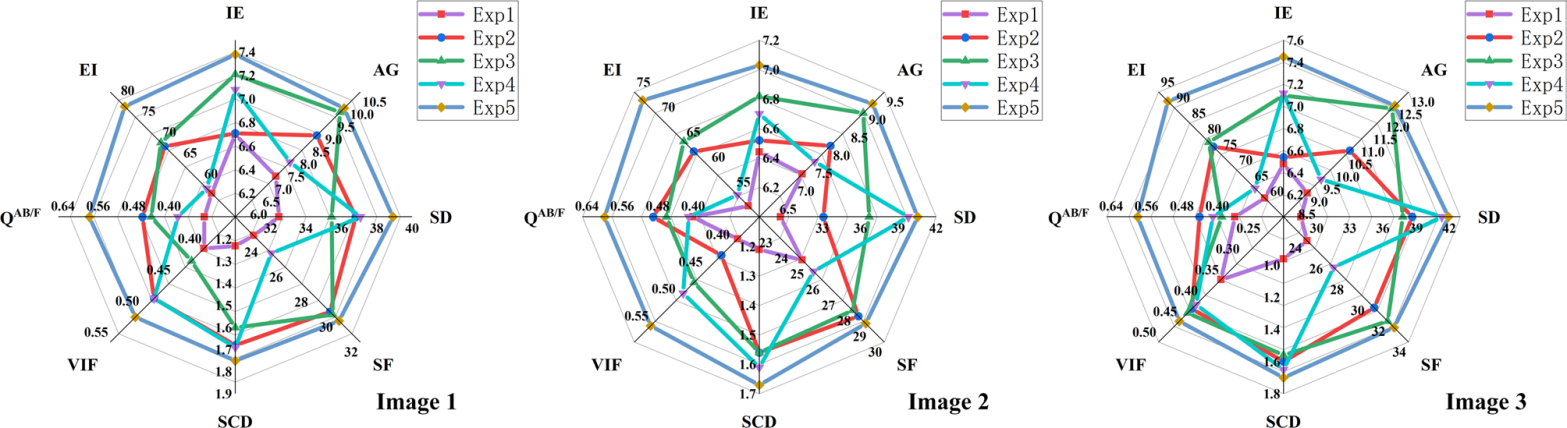
The ablation experiments evaluation metrics coefficient radar maps of CT mediastinal window images and PET images.

As shown in Table 6 and Fig. 13, The method in this paper is superior to the other 4 methods in 8 objective evaluation metrics, especially in AG, VIF, Q^AB/F^, and EI, the method in this paper has obvious advantages over the other 4 methods. In addition, the evaluation metrics value of Exp1 is the lowest. Exp3 and Exp4 have little difference with this paper in each evaluation metric. For example, Exp4 is only secondary to this method on SD, SCD, and VIF. Therefore, the fusion image obtained by the method in this paper has certain advantages in both subjective and objective evaluation.

**Figure 14:**
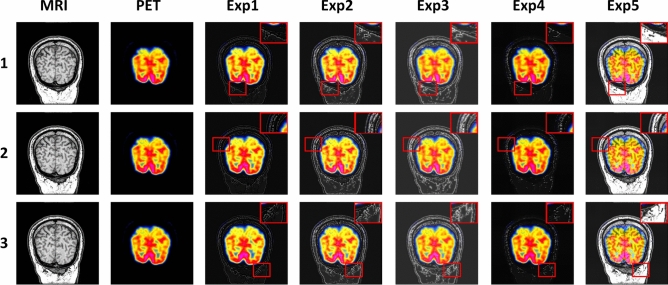
The ablation experiments fusion results of MRI brain images and PET images.

#### Ablation experiment 3: MRI brain image and PET image

As show in Fig. 14, the overall effect of Exp1 fusion images is poor and the contrast is low. The edge and texture information of the fusion image obtained by Exp2 are improved, but the brightness distribution is uneven. The fusion images obtained by Exp3 shows more prominent lesion areas, but blurred details. The fusion image obtained by Exp4 loses MRI structural information and has artifacts. The fusion image of Exp5 has the best performance in edge sharpness and contrast, and has rich details, and has a good visual effect.

**Figure 15:**
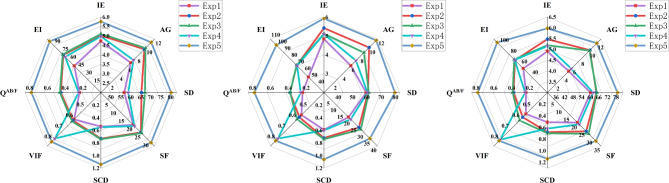
The ablation experiments evaluation metrics coefficient radar maps of MRI brain images and PET imagess.

As shown in Table 7 and Fig. 15, The method in this paper is superior to the other 4 methods in 8 objective evaluation metrics, especially in SD, VIF, Q^AB/F^, and EI, the method in this paper has obvious advantages over the other 4 methods. In addition, the evaluation metrics value of Exp1 is the lowest. Exp3 has little difference with this paper in each evaluation metric. Therefore, the fusion image obtained by the method in this paper has certain advantages in both subjective and objective evaluation.

**Table 7:** The ablation experiments evaluation metrics values of MRI brain images and PET images (Bold: best; Bolditalic: second best).

Image	Methods	IE	AG	SD	SF	SCD	VIF	${\textrm{Q}^{\textrm{AB}/\textrm{F}}}$	EI
1	Exp1	4.72	6.67	56.69	19.91	0.54	0.60	0.24	50.68
	Exp2	5.02	9.72	65.34	24.61	0.73	0.61	0.45	71.66
	Exp3	***5.10***	***9.98***	***67.08***	***25.01***	***0.74***	0.62	***0.46***	***72.78***
	Exp4	4.97	7.29	58.69	20.66	0.55	0.78	0.25	68.00
	Exp5	**5.75**	**11.27**	**80.20**	**30.89**	**1.14**	**0.81**	**0.78**	**97.73**
2	Exp1	4.83	6.02	59.54	18.40	0.59	0.56	0.24	53.11
	Exp2	5.41	***10.11***	60.86	26.08	0.72	0.58	***0.38***	70.64
	Exp3	***5.11***	8.96	***61.38***	***27.54***	***0.74***	***0.60***	0.38	***70.79***
	Exp4	5.09	7.17	60.46	19.97	0.60	0.78	0.26	70.10
	Exp5	**5.92**	**11.70**	**79.68**	**34.34**	**1.06**	**0.80**	**0.78**	**101.06**
3	Exp1	4.91	5.18	60.24	19.65	0.51	0.55	0.24	54.99
	Exp2	***5.47***	***10.31***	64.28	25.36	0.58	0.58	***0.38***	70.53
	Exp3	5.19	10.20	***64.61***	***26.94***	***0.60***	0.61	***0.39***	***71.83***
	Exp4	5.15	5.80	62.03	20.77	0.55	0.79	0.25	70.97
	Exp5	**5.97**	**12.00**	**79.06**	**31.69**	**1.14**	**0.80**	**0.78**	**103.67**

**Table 8:** FLOPs and parameters of LCFM for different ${\hat{F}}_{\text {temp}}$ sizes ($T{\times }T$).

${\hat{F}}_{\text {temp}}$ size	FLOPs (G)	Params (K)
$8{\times }8$	84.63	515.9
$32{\times }32$	135.24	720.4
$16{\times }16$ (Ours)	**94.75**	**556.8**

**Table 9:** Ablation experiment of ${\hat{F}}_{\text {temp}}$ sizes ($T{\times }T$).

${\hat{F}}_{\text {temp}}$ size	IE	AG	SD	SF	SCD	VIF	${\textrm{Q}^{\textrm{AB}/\textrm{F}}}$	EI
$8{\times }8$	7.39	12.41	41.56	31.93	1.66	0.47	0.58	91.77
$32{\times }32$	***7.41***	**12.46**	***41.79***	***32.11***	**1.71**	***0.49***	***0.61***	***91.86***
$16{\times }16$ (Ours)	**7.45**	***12.45***	**41.92**	**32.61**	***1.70***	**0.51**	**0.62**	**92.03**

#### Ablation experiment 4: ablation of pooling size

When calculating the correlation among modalities, we perform pooling operations on the feature map to reduce parameters and computational load. We selected three Pooling sizes, namely 8$\times$8, 16$\times$16 (Our), and 32$\times$32, and provided a comparison between the FLOPs/ parameter and the fusion metrics (IE, AG, SD, SF, SCD, VIF, QAB/F, EI). The experimental results are listed in Tables 8 and 9. When T=16, FLOPS and Parameters are only 94.75G / 556.8K; Compared with T=32, FLOPs decreased by 29.9%, parameters decreased by 22.7%, while the indicators were better or remained the same. Compared with T=8, although FLOPs increased by 12%, the metrics have been comprehensively improved. In conclusion, T=16 strikes the best balance between computational cost and fusion quality, so we set it as the default setting in the paper.

## Conclusion and future work

### Conclusion

Aiming at the existing multimodal medical image fusion methods ignore the feature dependence among modals, and the feature fusion ability with different granularity is not strong. This paper proposes a Long-Range Correlation-Guided Dual-Encoder Fusion Network for Medical Images. Firstly, a Long-Range Correlation-Guided Dual-Encoder Fusion Network for Medical Images is designed, which aggregates multi-scale features layer by layer and captures feature dependencies between modals, it achieves an effective fusion of different granularity features. Secondly, a Cross-dimension Multi-scale Feature Extraction Module is designed in the feature extraction stage, which effectively retains the coarse-to-fine grain features by extracting different scale information. Finally, the long-range correlation coefficients of local and global features are calculated by the Long-range Correlation Fusion Module, and the long-range dependencies between local and global features is captured. In addition, The method presented in this paper is validated on clinical multimodal lung medical image dataset and brain medical image dataset. On the lung medical image dataset, the evaluation metrics such as IE, AG, Q^AB/F^, and EI show average improvements of 4.53%, 4.10%, 6.19%, and 6.62%, respectively, compared to the optimal performance of the other 9 methods. On the brain medical image dataset, metrics like SF, VIF, and Q^AB/F^ show average improvements of 3.88%, 15.71%, and 7.99%, respectively, compared to the best performance of the other 6 methods. The experimental results show that the medical images fused by the model exhibit clear structures and rich texture details. This accomplishment provides valuable support for doctors’ diagnostic assistance and preoperative preparation.

### Future work

Although encoder–decoder network is widely used in the medical image fusion field. However, there are still some problems that need further study: Firstly, due to differences in imaging principles and dynamic organ deformation, most medical multimodal datasets have spatial registration errors; Secondly, the traditional method only relies on image information, but it lacks multi-source data integration (Such as medical history, doctor’s advice). Thirdly, the evaluation metrics of image fusion effect are not uniform, which leads to the lack of algorithm comparability. Therefore, the future encoder–decoder network research for multi-modal medical image fusion are further explored from the following directions: Firstly, combined with the cross-modal self-supervised registration method, which improves the accuracy and robustness of image registration. Secondly, Multi-source clinical data (such as medical history and doctor’s advice) are fused to enhance the model’s performance. Thirdly, a unified evaluation system is important to improve the algorithms comparability.

## Data Availability

1. The brain PET/MRI dataset used in this study is publicly available from the Harvard Brain Atlas: https://www.med.harvard.edu/AANLIB/home.html. 2. The clinical lung PET/CT dataset is not publicly available due to patient privacy restrictions but is available from the corresponding author upon reasonable request.
